# Impact of blindness due to cataract in elderly fallers: findings from a cross-sectional study in Andhra Pradesh, South India

**DOI:** 10.1186/s13104-018-3883-7

**Published:** 2018-10-29

**Authors:** Sannapaneni Krishnaiah, Ramani V. Ramanathan

**Affiliations:** Department of Community Outreach Services, Sankara Eye Foundation-India, Coimbatore, Tamil Nadu India

**Keywords:** Falls, Cataract surgery, Blindness, Southern India, Injury

## Abstract

**Objective:**

To estimate the prevalence of falls, frequency of falls, injury due to falls and to explore the relationship between cataract-related blindness and falls in older patients above or equal to 50 years of age.

**Results:**

A cross-sectional study was conducted to investigate the relationship between cataract related blindness and risk of fall. Details about any fall in the previous 12 months and systemic illness history were collected through a personal interview. Overall, 70 (18.3%; 95% confidence intervals (CI) 14.4%, 22.2%) of the 382 patients investigated had experienced falls. The history of recurrent falls were more commonly seen in patients with bilateral cataract (p = 0.023). The mean presenting Logarithm of the Minimum Angle of Resolution (LogMAR) visual acuity was significantly higher in fallers when compared to non-fallers: 0.81 ± 0.41 versus 0.65 ± 0.31 (p = 0.001). The prevalence of falls was significantly higher in patients with bilateral cataract blind; adjusted odds ratio (OR): 1.76 (p = 0.042). Timely diagnosis and surgical intervention in patients with bilateral blindness due to cataract may help prevent falls in older patients in Andhra Pradesh, South India.

## Introduction

Falls and fractures are common and serious preventable problems encountered by elderly population throughout the world and are an important cause of morbidity and mortality [1, 2]. Previously, it was estimated that close to 40% of healthy individuals over the age of 65 experience at least one fall a year [3]. Poor vision, reduced contrast sensitivity and depth perception were reported as important visual risk factors for falls in elderly [4–11]. A multidisciplinary review conducted previously had described the critical role of decreased visual acuity in fall events, particularly related to trips, slips and falls due to environmental hazards [12]. Health care costs as a result of fractures caused by falls are pervasive and substantial and correlate with the frequency and severity of falls [13].

Falls can be prevented through several evidence-based interventions. Identifying at-risk patients such as patients requiring cataract surgical intervention is an important part of management. Studies earlier have proved that cataract surgery on the first affected eye reduced falls in a population of older women [14].

However, there is a paucity of data on the association of cataract blindness and falls and falls with injury in the population of India. Few studies conducted previously in India have reported the prevalence of falls among elderly but not specific to the cause of blindness [15–18]. The present study seeks to evaluate the relationship between cataract related blindness and risk of falls and injury due to falls among elderly in the state of Andhra Pradesh, Southern India.

## Main text

### Methods

A hospital-based, cross-sectional study was conducted in the Guntur district of Andhra Pradesh, South India during December 2016 to March 2017 among patients aged 50 years and above diagnosed with either unilateral or bilateral operable cataract as part of the community outreach program offered by a Tertiary Eye Hospital. A total of 401 patients requiring cataract surgical services were randomly selected and invited to participate in the study. Of these, complete data were available for 382 (95.3%) patients who responded to a structured questionnaire. The socioeconomic status of the patient was determined by his or her ability to pay for surgery. Informed consent was obtained from all participants before interview either by thumb impression or signed by the participant. The study was approved by the Institutional Review Board of Sankara Eye Hospital, Bengaluru. The study adhered to the tenets of the Helsinki Declaration.

Sample size was calculated using the prevalence estimate of falls in visually impaired population among elderly reported as 52.4% previously in India [18]. Considering this as the baseline estimate and at 5% level of significance and relative precision respectively, the sample size estimated was 383. Assuming further that 10% of the patients may not respond to interview, a total of 421 were estimated.

This study included both men and women aged ≥ 50 years, able to move indoors without walking aids and on examination were confirmed to be operable for unilateral or bilateral cataract graded post dilation as nuclear cataract grade 5 to grade 1. Patients with other cataracts included were cortical and posterior subscapular opacities. Patients with retinal diseases, glaucoma and corneal disease were excluded. The demographic details and history of hypertension and/or diabetes including history of using medication were elicited. Diastolic and Systemic blood pressure was measured by sphygmomanometry in an upright sitting position after a 5 min rest period. Patients whose blood pressure reading was ≥ 140/90 mm Hg and/or on medication for control of hypertension were considered to be hypertensive. Presence of diabetes was confirmed by using the fasting plasma glucose test and/or on medication for control of diabetes. An individual patient who can pay for cataract surgery was considered to be middle and upper socioeconomic category group. Data with respect to history of falls were elicited from participants to include falls where any part of the body above the ankle hit the floor or ground and falls which occurred on stairs and during the work in work place and was defined as unintentionally coming to the ground or some lower level and not as a result of a major intrinsic event (e.g. stroke) or overwhelming hazard [19]. Details regarding recurrent falls and injuries resulting from any fall were elicited. A participant was classified as a “faller” if a fall had occurred in the past 12 months. The selective clinical parameters were extracted from the case sheets of the each patient registered for cataract surgery in the hospital. The investigator who administered the questionnaire was masked to the visual acuity and comorbidity details of the patients.

### Statistical analysis

Data were initially explored through descriptive statistics. Univariable associations between various risk factors and falls with potential covariates were analysed by using the χ^2^ test and the extent of risk was expressed using the logistic regression model. Further to find out risk factors associated with the fall, multivariable logistic regression model was employed adjusting for the potential confounders. The adjusted ORs with 95% CI were also derived for the possible interaction effects in the model whose fitness was assessed by using the Hosmer–Lemeshow goodness-of-fit test statistics, in which p > 0.05 indicates no evidence of misfit. In addition, separate linear regression models were applied to study the relationship between number of falls and visual acuity in LogMAR units in the better eye. A two sided p < 0.05 was considered to be statistically significant. All statistical analysis were conducted using the Statistical Package for Social Sciences (SPSS) version 17.0 (SPSS, Chicago, IL, USA) for Windows.

## Results

The sociodemographic and clinical characteristics of the fallers and non-fallers are shown in Table 1. Overall, the mean age was 63.9 ± 6.9 years; range: 50–89 years and there were 202 (52.9%) females. A total of 111 (29.1%) patients reported a history of diabetes and hypertension with an evidence of using medication. A majority (63.9%; n = 71) of them belonged to working status group. Overall, there were 107 (28.0%) (Best corrected distance LogMAR visual acuity (BCVA) < 6/60 in the better eye) and 14 (3.7%) (BCVA < 3/60 in the better eye) patients blind according to Indian and World Health Organization’s definitions respectively.

**Table 1:** Characteristics of the study participants by fallers and non-fallers (N = 382)

S no	Characteristics	Category	Fallers (n = 70)	Non-fallers (n = 312)	p value
1	Gender	Male	29 (41.4)	151 (48.4)	
		Female	41 (58.6)	161 (51.6)	0.354
2	Age (years)		64.9 ± 5.7	63.7 ± 7.2	0.141
3	Education	Illiterate	39 (55.7)	148 (47.6)	
		School	26 (37.1)	143 (45.9)	
		College	5 (7.1)	20 (6.4)	0.403
4	Socioeconomic status^a^	Lower and extreme lower	68 (98.6)	280 (90.6)	
		Middle and upper	1 (1.4)	29 (9.4)	0.025
5	Occupation	Working (agriculture labor/daily wage laborer/clerk/watchman/own business/plumber/vegetable seller)	49 (70.0)	195 (62.5)	
		Retired/housemaker/non-working	21 (30)	117 (37.5)	0.272
6	Systemic illness	Yes	28 (40.0)	83 (26.6)	
		No	42 (60.0)	229 (73.4)	0.029
7	Vision status^b^	<6/6 to 6/18	11 (15.9)	72 (23.3)	
		<6/18 to 6/60	28 (40.6)	146 (47.2)	
		<6/60 to 3/60	23 (33.3)	84 (27.2)	
		<3/60	7 (10.1)	7 (2.3)	0.007
8	Eye operated	Unilateral	38 (54.3)	217 (69.6)	
		Bilateral	31 (44.3)	93 (29.8)	0.023

^a^Data on socioeconomic status was not available for 4 participants

^b^Data was not available on 4 patients

Overall, 70 (18.3%; 95% CI 14.4%, 22.2%) patients had history of at least one fall during the past 12 months. History of falls were more commonly seen in patients with bilateral cataract (Table 1). Falls in the past 12 months were reported in 29 males and 41 females. Among these, fall recurrence occurred in 24.3% (n = 17) fallers. Number of falls ranged from 1 to 7 multiple times. Fall led to injury in 25.7% (n = 18) patients. Fall was situated at home in 10.0% (n = 7) patients or outside at work place in 88.6% (n = 62) patients.

**Fig. 1:**
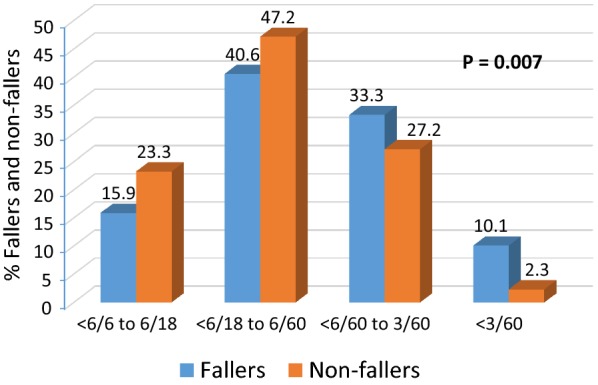
Proportion of participants who suffered falls and non-fallers classified with respect to visual acuity

Table 2 reports the results of univariable and multivariable logistic regression analysis. Patients with history of systemic illness (diabetes or hypertension) had significantly higher prevalence of falls; adjusted odds ratio (OR): 2.06 (95% CI 1.17, 3.62) (p = 0.012) (Table 2). Patients categorized blind as per Indian definition had significantly higher prevalence of falls; OR: 1.76 (95% CI 1.02, 3.04) (p = 0.042). The prevalence was fivefolds higher in patients defined as blind according to the WHO definition (Table 2). The prevalence of falls was higher in those belonging to lower and extreme poor socioeconomic group, however, it was borderline significant (p = 0.051). Decreased visual acuity was significantly associated with increased risk of falls (p = 0.007) (Fig. 1).

**Table 2:** Univariable and multivariable logistic regression analysis of risk factors for falls (N = 382)

Factors	Total sample	Fall n (%)	Unadjusted OR (95% CI)	p value	Adjusted OR (95% CI)^b^	p value
Age (years)						
< 60	79	11 (13.9)	1.00		1.00	
≥ 60	303	59 (19.5)	1.50 (0.75, 3.01)	0.259	1.30 (0.61, 2.74)	0.495
Gender						
Male	180	29 (16.1)	1.00		1.00	
Female	202	41 (20.3)	1.33 (0.79, 2.24)	0.292	1.25 (0.71, 2.21)	0.439
Education status						
Literate	194	31 (16.0)	1.00		1.00	
Illiterate	187	39 (20.9)	1.39 (0.82, 2.33)	0.220	1.03 (0.58, 1.84)	0.921
Economic status						
Middle and upper	30	1 (3.3)	1.00		1.00	
Lower and extremely poor	348	68 (19.5)	7.04 (0.94, 52.62)	0.057	7.85 (0.99, 62.09)	0.051
Systemic illness						
No	271	42 (15.5)	1.00		1.00	
Yes	111	28 (25.2)	1.84 (1.07, 3.16)	0.027	2.06 (1.17, 3.62)	0.012
Blindness (Indian definition)						
≥ 6/60	255	38 (14.9)	1.00		1.00	
< 6/60	124	31 (25.0)	1.90 (1.12, 3.24)	0.018	1.76 (1.02, 3.04)^b^	0.042
Blindness (WHO definition)^a^						
≥ 3/60	363	61 (16.8)	1.00		1.00	
< 3/60	15	8 (53.3)	5.66 (1.98, 16.18)	0.001	5.98 (1.99, 17.98)	0.001

Unadjusted OR, unadjusted odds ratio; adjusted OR, adjusted odds ratio; 95% CI, 95% confidence intervals; WHO, World Health Organization

^a^Replaced in the logistic regression model

^b^The Hosmer–Lemeshow test statistics indicates good fit of the model: χ^2^ = 8.804 and p = 0.359

According to the linear regression model, for each unit increase in the LogMAR visual acuity in the better eye, there were 0.55 units (Beta coefficient = 0.55; p < 0.001) increase in the number of falls. We also explored the associations between falls and interaction of systemic illness and blindness in the model. The analysis revealed a significant association between falls and interaction term of systemic illness and blindness inputted as a combined effect; adjusted OR: 2.49 (95% CI 1.43, 4.34) (p = 0.001).

## Discussion

There is a paucity of data reporting the associations between visual impairment and falls and injuries due to falls in Indian population. In this study, we found an evidence of the relationship between cataract related blindness and falls. Further, the study revealed an evidence of an association between presence of systemic illness (diabetes/hypertension) and falls which is in accordance with previously published reports [20]. Patients with severe visual impairment (Presenting Visual Acuity (PVA)) < 3/60 in the better eye) showed an increased prevalence of falling by more than fivefold, although our findings must be treated with caution, considering small number of patients studied in this category of blindness. Overall, our findings substantiate previous studies confirming that severe visual impairment is a significant risk factor for falls and injuries resulting from falls [5, 11, 12, 21–25].

The prevalence of falls in our study was 18.3% which is in accordance to the prevalence estimates reported previously, the Longitudinal Aging Study Amsterdam (16.2%) [21], the Study of Osteoporotic Fractures (16.4%) [5]. Our study has demonstrated two important findings which are similar to the findings published previously [5, 21, 22], namely (i) Decrease in visual acuity was significantly correlated with increased risk of falls (Fig. 1); (ii) Change in visual acuity (LogMAR) has significantly predicted the occurrence of frequent falls suggesting that correction of visual loss helps to reduce the future falls and its associated burden in the community. A study conducted previously in hospitalized patients revealed a significant association between visual impairments and falls (adjusted odds ratio: 13.9%; p < 0.001) which is in accordance with our study findings [22]. Our study findings demonstrate the importance of cataract surgical intervention in elderly patients in order to prevent the falls due to decreased visual acuity which support the evidence published most recently by Stephen Lord and others [10, 11, 23, 24]. Reduced ability to detect low contrast hazards, judge distances and perceive spatial relationships by patients are the major visual impairment associated with increased risk of falls [11]. In addition to it, the multifocal glasses may add up to the risk of falls in elderly patients due to their reduced ability to detect environmental hazards, therefore, it is also an important criteria for ophthalmologists and health care professionals to consider contrast sensitivity measures when prioritizing cataract patients for surgery [5, 11].

The prevalence of falls reported most recently from The Singapore Malay Eye Study was 14.7% which is slightly less than the estimate from current study [26]. However, the Salisbury Eye Evaluation (SEE) reported a higher estimate of falls 29% [7]. The SEE Project investigated participants who were considerably older than our study participants (75 years vs 64 years) which might have caused the differences in prevalence estimates. Similar or even higher prevalence estimates of falls ranging from 28 to 35% have been reported earlier in elderly population [2, 27–29]. However, from the time that these studies were conducted there has been medical advancement in care available to the patients that might be the reason for the reduced prevalence of falls noticed in this present study. Although, earlier studies conducted in India have found prevalence of falls to range from 14 to 53%, however, these data are not vision specific for comparisons with our study findings [30–32].

Linear regression analysis of this study data revealed a significant relation between LogMAR visual acuity and number of falls (b = 0.55; p < 0.001), which is in accordance with published reports [33]. Association between systemic illness and blindness and risk of falls have clinical implications for clearing the backlog of cataract burden as well as to implement effective strategies to deal with surgery related complications which is supported by published evidence that highlights “cataract surgery can prevent falls” [34–36].

## Conclusions

In summary, this study documents the prevalence of falls and its association with blindness in the Southern Indian population. To our knowledge, this is the first of its kind to document the associations between visual impairment due to cataract and risk of falls, frequency of falls and injuries due to falls in the Indian population. These results can be helpful for future research and planning effective strategies to prevent falls among elderly population. The existing community eye health initiatives should be strengthened that focuses to raise awareness especially in older population and their caregivers of the importance of regular eye examinations and use of appropriate prescription glasses for prevention of falls [11]. In addition, this study highlights the importance of screening among older adults for operable cataract and identification of those at risk of future falls in order to take precautionary steps thereby improving the quality of life of patients.

## Limitations

The limitation of the study was the relatively small sample size and future studies should include larger representative sample with varying level of visual impairment from not only cataract but also from diabetic retinopathy, glaucoma and age-related macular degeneration. Nevertheless, these findings suggest that decreased visual acuity due to cataract is the most important predictor of increased prevalence of falls and falls with injuries in this population.

## Abbreviations

LogMAR: Logarithm of Minimum Angle of Resolution
CI: confidence intervals
OR: odds ratio
SPSS: Statistical Package for Social Sciences
BCVA: best corrected distance visual acuity
PVA: presenting visual acuity
SEE: Salisbury Eye Evaluation
